# *In-Situ* Welding Carbon Nanotubes into a Porous Solid with Super-High Compressive Strength and Fatigue Resistance

**DOI:** 10.1038/srep11336

**Published:** 2015-06-11

**Authors:** Zhiqiang Lin, Xuchun Gui, Qiming Gan, Wenjun Chen, Xiaoping Cheng, Ming Liu, Yuan Zhu, Yanbing Yang, Anyuan Cao, Zikang Tang

**Affiliations:** 1 State Key Lab of Optoelectronic Materials and Technologies, School of Physics and Engineering, Sun Yat-sen University, Guangzhou, 510275, P. R. China; 2 Department of Materials Science and Engineering, College of Engineering, Peking University, Beijing 100871, P. R. China; 3 Department of Physics, Hong Kong University of Science and Technology, Clear Water Bay, Kowloon, Hong Kong, China

## Abstract

Carbon nanotube (CNT) and graphene-based sponges and aerogels have an isotropic porous structure and their mechanical strength and stability are relatively lower. Here, we present a junction-welding approach to fabricate porous CNT solids in which all CNTs are coated and welded *in situ* by an amorphous carbon layer, forming an integral three-dimensional scaffold with fixed joints. The resulting CNT solids are robust, yet still highly porous and compressible, with compressive strengths up to 72 MPa, flexural strengths up to 33 MPa, and fatigue resistance (recovery after 100,000 large-strain compression cycles at high frequency). Significant enhancement of mechanical properties is attributed to the welding-induced interconnection and reinforcement of structural units, and synergistic effects stemming from the core-shell microstructures consisting of a flexible CNT framework and a rigid amorphous carbon shell. Our results provide a simple and effective method to manufacture high-strength porous materials by nanoscale welding.

Carbon-based porous materials have numerous applications in energy, environmental and other fields^1^,^2^,^3^,^4^,^5^. Recently, carbon nanotubes (CNTs) and graphene have proven to be promising structural units for manufacturing three-dimensional (3D) macroscopic materials with low-density and high porosity *via* bottom-up approaches^6^,^7^,^8^. Typical methods include chemical vapor deposition (CVD), critical point drying and freeze casting, which have resulted in a variety of fascinating structures such as CNT and graphene-based aerogels, foams and sponges^9^,^10^,^11^,^12^. Although individual CNTs and graphene are intrinsically stiff and strong, the mechanical properties of their self-assembled porous networks are primarily limited by the microscale morphology and interaction between structural units. Actually, vertically aligned CNT arrays, an anisotropic porous structure, have been studied extensively and exhibited attractive mechanical properties. Under axial compression, the CNT arrays behave rather strong, with compressive moduli ranging from several MPa to tens of GPa, depending on the synthesis techniques, the resulting CNT diameter and alignment^13^,^14^,^15^,^16^,^17^,^18^,^19^,^20^. In particular, coating CNT arrays with a-SiC, Al2O3 and amorphous carbon could significantly enhance the compressive moduli to very high values (25–125 GPa), as reported recently^21^,^22^,^23^. On the other hand, isotropically configured CNT aerogels and sponges (especially those with high porosity) are very soft (with compressive strengths on the order of kPa) compared with aligned arrays, and readily produce permanent (plastic) deformation under cyclic loading conditions^3^,^6^,^9^,^24^. For these random structures, many methods have been developed to modify the porous structure by forming covalent junctions^25^, nitrogen doping^26^, or introducing foreign molecules (e.g. polymer, graphene, amorphous carbon)^8^,^27^,^28^, however, the resulting materials are not strong and the effect of reinforcement is rather limited.

Welding, with its tradition that can be traced back to the Bronze and Iron Ages, is an important technique for joining metals or remediating structural defects. Nanoscale features may also be assembled by downsizing this technique. Indeed, occasional welding or merging of some nanomaterials (e.g. nanoparticles, nanowires, nanotubes, nanofibers) has been observed under particular conditions such as heating, irradiation, etc^29^,^30^,^31^,^32^. Such welding phenomena mainly occur at individual level, and formation of a large-scale, integral structure remains a big challenge. In CNT and graphene aerogels, nanoscale units are ordinarily assembled *via* van der Waals forces or π-π attractions, resulting in weak molecular interactions, and slipping at contact points is one of the major reasons for permanent deformation^3^,^12^,^24^. In this regard, welding could be an effective approach to stabilize the contact points and prevent slipping. Monthioux, *et al.* have reported the deposition of pyrolytic carbon on CNTs to form different structures^33^,^34^,^35^. Recently, we also deposited amorphous carbon onto a CNT sponge and observed improved elasticity due to the thin amorphous coating on CNTs and their junctions, although the compressive strength remains low (<1 MPa)^27^.

Here, we report the synthesis of monolithic CNT-based solids in which all CNTs throughout the bulk are embedded within an amorphous coating and welded at their contact points *in situ* during a CVD process; the resulting structure is in analogous to a nanoscale 3D scaffold with fixed joints. Compared to previous two-step (synthesis and welding) coating method, our *in situ* (one step) synthesis processes are simple, and considerably different in terms of material structures and resulting mechanical properties. Owing to the significant reinforcement by simultaneously thickening and welding nanotube skeletons, the CNT solids show tailored compressive strengths up to 72 MPa, high flexural strengths (20–33 MPa) and fatigue resistance (recovery after 100,000 large-strain compression cycles); these mechanical properties are distinct from previous CNT and graphene-based porous materials. The compressive strength of our CNT solids is close to commercial graphite products, yet still possessing high porosity and compressibility. Nanoscale welding is thus a potential route to overcome the weak molecular interactions existing in a wide range of self-assembled nanostructures and achieve high-strength porous materials.

## Results

**Figure 1:**
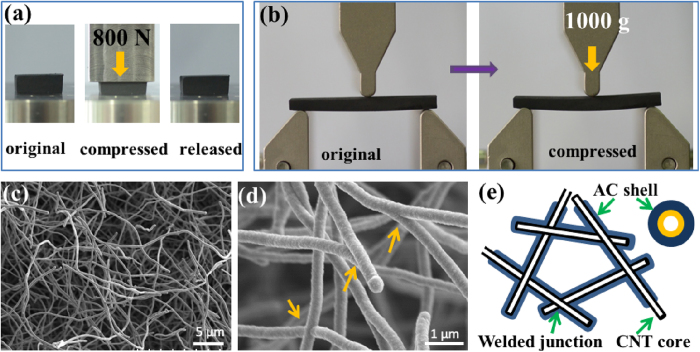
Strong carbon nanotube solids with welded scaffold. (**a**) Photos of a CNT solid (10 × 10 × 4 mm^3^) compressed by a load of 800 N (2 MPa) with its thickness reduced by 30%, and recovered to original shape after compression. (**b**) A CNT solid being subjected to three-point bending under a weight of 1000 g (1 MPa) without breaking (sample size 35 × 9 × 2.5 mm^3^). (**c**) and (d) SEM images of the CNT solid, revealing a porous structure with many welded junctions (see arrows). The CNT solids in (**a**)–(**d**) were synthesized at a dichlorobenzene supply rate of 0.5 mL/min and methane flowing rate of 30 sccm. (**e**) Illustration of the core-shell structure of AC coated and welded CNT scaffold.

*In situ* welding of CNTs was carried out by a modified CVD process previously used for synthesizing CNT sponges^3^. Here, we supplied two carbon sources by injecting liquid dichlorobenzene and flowing gaseous methane into the CVD furnace at the same time (see Experimental for details). When only dichlorobenzene was injected at a lower rate (0.1 mL/min), clean CNT sponges were obtained. When the feeding rate increased to 0.3–1.0 mL/min and methane was introduced as well, CNTs and amorphous carbon (AC) grew simultaneously due to over-supply of carbon source. The effect of methane is to facilitate uniform deposition of amorphous carbon throughout the sponge and form smooth coatings on CNTs. The resulting samples (so called CNT solids) feel very hard during manipulation, completely different from pure CNT sponges which are soft and easily deformable. Under a load of 800 N (the loads as large as 1 × 10^6^ times of its own weight), the solid reduced its thickness by 30% and then recovered to original volume when the load was released (Fig. 1a). This result indicates that the CNT solid is super-strong yet still compressible. Furthermore, the material is so hard that it can withstand three-point bending at high loading (1000 g), only producing a slight curvature (Fig. 1b). Conventional CNT and graphene aerogels/foams are too soft to resist this type of bending^3^.

The above observations imply that the material structure has changed fundamentally. Scanning electron microscopy (SEM) characterization on the inner part of the sample reveals a porous network with many welded junctions; these fibers are amorphous carbon-coated CNTs (AC-CNTs) with a core-shell structure (Fig. 1c). In a small area, junctions with different shapes such as “∧”, “Y”, “+” are easily found (Fig. 1d). Simultaneous growth of amorphous carbon during CVD leads to the welding of CNTs at their contact points and formation of an integral 3D scaffold with high strength. Now the basic units become the AC-coated CNTs, in which the AC layer covers uniformly throughout the CNT framework and serves as a promoter for nanoscale welding (the microstructure is illustrated in Fig. 1e). All contact points through the CNT network have been converted to welded junctions. This structure is entirely different from other CNT-based macrostructures where CNTs are assembled through van der Waals forces^7^.

**Figure 2:**
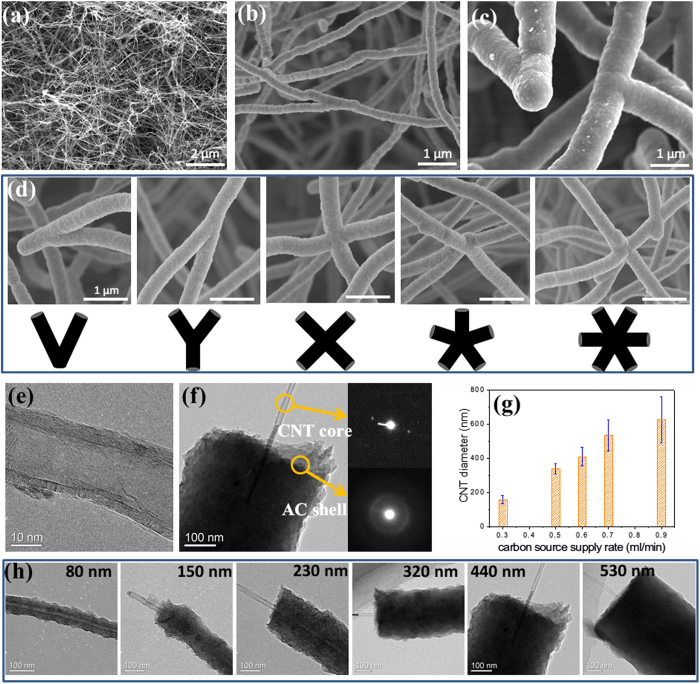
Microstructure characterization of AC-welded CNT solids. (**a**)–(**c**) SEM images of a CNT solid synthesized using a dichlorobenzene supply rate of 0.1, 0.3 and 0.7 mL/min (methane flowing rate set as 30 sccm), respectively. The CNT diameters increase rapidly with more carbon source. (**d**) A collection of SEM images of welded junctions with different numbers of CNTs. Inset: illustration the welded junction structures. (**e**) TEM image of a CNT without AC coating. (**f**) TEM image of a CNT coated by AC (thickness of ∼200 nm), and corresponding selected area electron diffraction patterns of the CNT core and AC shell. (g) CNT diameters controlled by different dichlorobenzene supply rates. (h) TEM images of core-shell AC-CNT structures with different AC thicknesses. A single CNT protrudes out from the ends.

The microstructure of the CNT solids can be tailored by the feeding rate of carbon sources. At a lower dichlorobenzene injection rate (0.1 mL/min) which is a typical parameter for synthesizing CNT sponges, the network consists of pure CNTs with diameters of 30–50 nm; there is no AC deposition on CNT surface (Fig. 2a). At higher feeding rate (0.3 to 0.9 mL/min), AC-coated CNTs starts to grow and thicker fibers with welded junctions are observed within the solid (Figs. 2b,c, and Fig. S1). The AC deposition and welding of CNTs are very efficient processes, resulting in numerous inter-CNT junctions with various morphologies (such as ∨, Y, ×, *, structures connecting 2 to 6 nanotubes) throughout the internal of the solid (Fig. 2d). Transmission electron microscopy (TEM) images clearly show the seamless wielding of the junctions (Fig. S2). The TEM images also clearly reveal the core-shell structure in which a multi-walled nanotube is wrapped by a thick AC shell with a seamless interface (Figs 2e,f). Selected area electron diffraction characterization reveals the amorphous nature of the AC shell, which produces a ring pattern (Inset of Fig. 2f). Also, Raman spectra of the sample with different densities show high defective bands (Fig. S3).

The core-shell structure indicates that the CNT network serves as the template for AC deposition during CVD, which ensures the formation a 3D network. At the same time, the AC coating facilitates the welding of CNTs at their contact points. In the solids, the CNTs overlap randomly creating many configurations such as end-to-end, end-to-body, and cross-junctions by two or multiple nanotubes; these are potential places to become welded joints during subsequent AC deposition. Correspondingly, welded contacts with different morphologies (as seen in Fig. 2d) form when AC deposits smoothly and continuously onto these regions.

In addition to welding the inter-CNT contacts, AC deposition also results in nanotube thickening. Upon increased dichlorobenzene feeding rate, the average outer diameter of AC-coated CNTs increases continuously from about 157 nm (at 0.3 mL/min) to 624 nm (at 0.9 mL/min) (Fig. 2g). The maximum AC shell thickness reaches 290 nm, much larger than the CNT diameter (30–50 nm). A series of core-shell structures with different AC layer thicknesses can be clearly viewed in TEM images; all structures have a single CNT as the inner core (Fig. 2h). Furthermore, changing the second carbon source (CH_4_) flowing rate also could tailor of the AC thickness and outer diameter within a certain range (∼260 to 400 nm) (Fig. S4).

With more AC deposition, the apparent bulk density of the CNT solid also increases consistently and reaches ∼82 to 573 mg/cm^3^ when the carbon source supply rate was increased from 0.3 mL/min to 0.7 mL/min (Fig. S5). At this density range, the BET surface area is about 20–30 m^2^/g; and the solids still retain a high porosity (75 ∼ 96%) and also compressible. Our CNT solids are much denser than other porous structures including CNT sponges (5–25 mg/cm^3^)^3^,^24^, graphene aerogels (1–200 mg/cm^3^)^36^, vertically aligned CNT arrays (8–210 mg/cm^3^)^37^,^38^,^39^, and even comparable to densified CNT films (470–980 mg/cm^3^)^40^.

**Figure 3:**
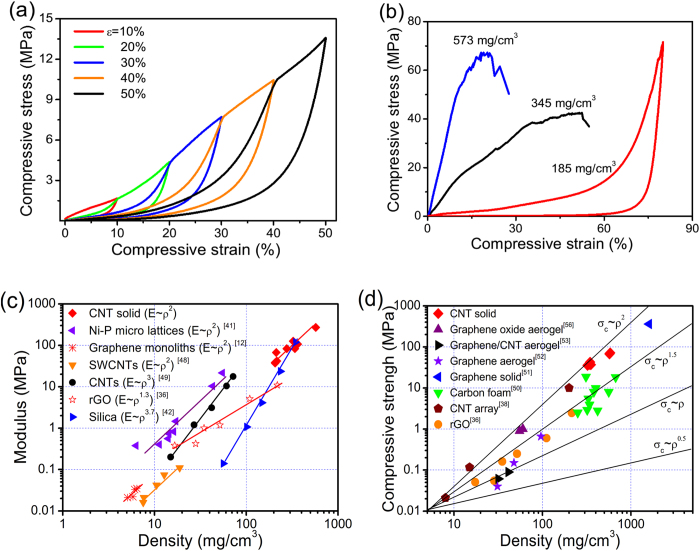
Mechanical properties of the CNT solids. (**a**) Typical compressive stress-strain (σ-ε) curves of CNT solids tested to different maximum strains. (**b**) Compressive curves for the samples with different densities. It shows a transition from ductile to brittle with the increase of density. (**c**) Classical Ashby materials selection map comparing mechanical properties of CNT solids grown in this study with those of several carbon-based porous materials reported in the literature. The modulus (*E*) of these CNT solids showed a power law dependence of *E* ∝ *ρ*^*2*^ on density (*ρ*), which further confirmed the open cell structure of the CNT solids. (**d**) Compressive strength (*σ*_*c*_) versus density *ρ* for CNT solids, and other carbon-based porous materials. *σ*_*c*_ values of our CNT solids at high density were larger than *σ*_*c*_ values of other reported carbon-based porous materials. The guidelines indicate the structural efficiency of the material. The data in (**c**) and (**d**) obtained form^12^,^36^,^38^,^41^,^42^,^48^,^49^,^50^,^51^,^52^,^53^,^54^,^55^,^56^,^57^.

We have performed systematic mechanical tests on the CNT solids. A solid sample (with density of 210 mg/cm^3^) was compressed to a series of strains (ε = 10% to 50%) and then released (Fig. 3a). The compressive stress (σ) reaches 13.6 MPa at ε = 50%, which is about three orders of magnitude higher than that of CNT with no AC welding (38 kPa)^3^, and graphene aerogels (7 kPa)^12^. The sample recovers original thickness during unloading, indicating excellent recoverability. Stress degradation also occurs during the second and following loading cycles due to fracture of the AC shell (characterized later) under compression. This behavior has been observed in porous metallic structures and ceramic materials with a brittle skeleton^41^,^42^,^43^.

The mechanical properties are dependent on the bulk density of CNT solids. A solid with lower density (185 mg/cm^3^) can be compressed to very high strain and stress (ε = 80%, σ = 71.6 MPa) without fracture, and can return to original thickness upon unloading (Fig. 3b). In comparison, a higher density sample (345 mg/cm^3^) collapses at a compressive strain of 52% (Fig. S6). A 573 mg/cm^3^ density sample shows a compressive strength of 67 MPa (maximum point in the σ-ε curve), a modulus of ∼270 MPa in the initial linear region, and can sustain a compressive strain of >20% before fracture (Fig. 3b). These strength (67 MPa) and modulus (270 MPa) values are enhanced by up to 4 orders of magnitude from original CNT sponges with a random microstructure^3^. However, compared with CNT arrays reinforced by Al_2_O_3_ or a-SiC coating in which the modulus has reached 20 GPa (CNT-Al_2_O_3_ arrays) and 125 GPa (CNT-a-SiC arrays), our AC-CNT sponges remain much weaker^21^,^22^. This difference is due to the following three factors including: 1) pristine CNT arrays are generally stronger than CNT sponges, giving a higher initial modulus; 2) the aligned structure results in higher stiffness under axial compression than randomly stacked CNTs; and 3) the bulk densities of Al_2_O_3_ or a-SiC coated CNT arrays (∼1000 mg/cm^3^) are also higher than AC-CNT sponges. With increasing density and AC coating thickness, mechanical behavior gradually shifts from ductile (highly compressible) to more like brittle materials with higher modulus and reduced failure strain. The density and AC coating thickness are about 345 mg/cm^3^ and 184 nm for this ductile-brittle transition, respectively. There are two underlying factors related to the unique structure of our CNT solids that lead to those superior properties. First, the AC coating provides a rigid support on CNTs (which are very flexible) and enhances compressive strength; without AC, the CNTs tend to buckle under compression. Second, the CNT scaffold with perfectly welded junctions ensures high structural stability, recovery and compressive strength (slipping between CNTs is inhibited). Welding is the most important factor resulting in the enhanced compressive strength and fatigue resistance. Consequently, our AC-reinforced CNT solids integrate very high strength, appreciable compressibility and high recoverability, and this combination has been rarely found in other porous materials. The CNT solid also strongly resists bending, resulting in a flexural strengths up to 33.4 MPa during 3-point bending tests (Fig. S7). Although this flexural strength is still lower than some ceramic materials such as glass and Al_2_O_3_, it is among the highest reported for porous carbon materials, compared with rubber, polymer foams^44^,^45^,^46^.

The compressive modulus (*E*) of CNT solids scales with the apparent density (*ρ*) in a squared relationship as *E* *∼* *ρ*^*2*^ (Fig. 3c), a behavior usually found in open-cell structures although the CNT solids have much higher density^12^,^41^. Similar modulus-density relationship has been observed in other low-density carbon-based nanomaterials, silica aerogels and nickel metallic lattices^12^,^41^,^42^. The compression strength (*σ*_*c*_) of CNT solids (up to 72 MPa, sample density of 582 mg/cm^3^) are comparable to conventional graphite products and isostatically pressed graphite, but the density of our porous CNT solids is only 25% of graphite^44^. A summary of material modulus (and strength) versus bulk density for a bunch of relevant materials is plotted in Fig. 3c,d.

**Figure 4:**
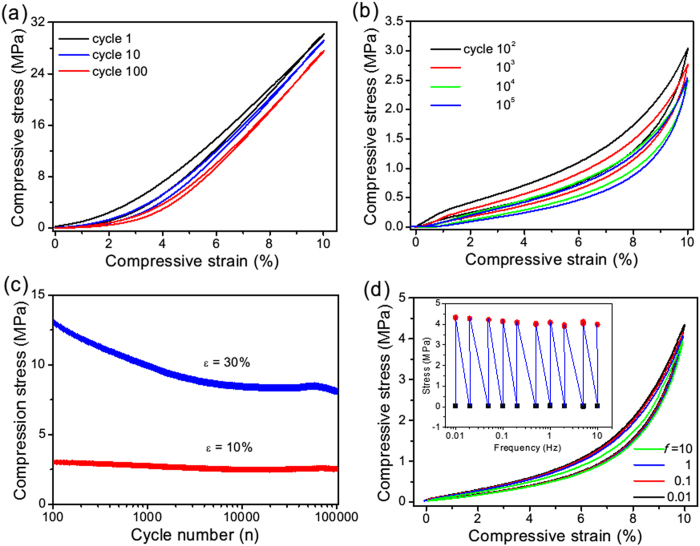
Fatigue resistance and frequency independence of the CNT solids. (**a**) σ-ε curves for the CNT solid with a density of 570 mg/cm^3^ (CNT diameter about 534 nm), measured after pre-compression and showing a narrow σ-ε hysteresis loop, implying super-elastic characters. (**b**) Selected σ-ε curves for the CNT solid over 1 × 10^5^ cycles compression. (**c**) Recorded compressive stress at different set strains of 10% and 30%, respectively. (**d**) σ-ε curves of the CNT solids compressed under different frequencies from 0.01 up to 10 Hz. No significant change in the stress responses and hysteresis loops was observed over the entire compression frequency range of three orders of magnitude. Inset: recorded compressive stress at different set frequencies.

Structural reinforcement is also reflected by the fatigue resistance of CNT solids during cyclic tests. Pre-compression could remove the stress degradation in initial loading cycles, and *σ-ε* curves for the following 100 cycles show very narrow hysteresis loops (Fig. 4a), in contrast to typical porous materials producing large stress loops^36^,^41^,^47^. This difference indicates that the energy dissipation is strongly inhibited in the CNT solids, since the AC coating and welding of CNTs could remove the zipping-unzipping process which is the main mechanism for dissipating energy during deformation^24^. The AC-welded structure is so stable that the CNT solids can sustain 1 × 10^5^ cycles (ε = 10% at 1 Hz) with negligible residual strain (<1%), indicting high fatigue resistance (Fig. 4b). In comparison, polymeric foams and CNT sponges show much larger plastic deformation (10%–30%) after hundreds of cycles^3^,^44^. The maximum stress at ε = 10% remains stable during repeated compression; when the solid is compressed to a larger strain (ε = 30%), the corresponding maximum stress decreases from 13 to 8 MPa after 10^5^ cycles (Fig. 4c).

Since the CNT solids show excellent recoverability during compression, the question is how fast the solids could expand back to original thickness (volume) from the compressed state? To investigate this, the samples were tested under a frequency range from 0.01 Hz (nearly static) to 10 Hz (dynamic). During testing, the samples can keep up with the movement of compression stages without any lagging. Nearly identical *σ-ε* curves and maximum stresses were obtained at those frequencies (Fig. 4d and inset), indicating very fast recovery of CNT solids which is useful for applications in high-frequency dynamic conditions. Such frequency-independent behavior is distinct from conventional polymer and rubber foams^44^.

## Discussion

**Figure 5:**
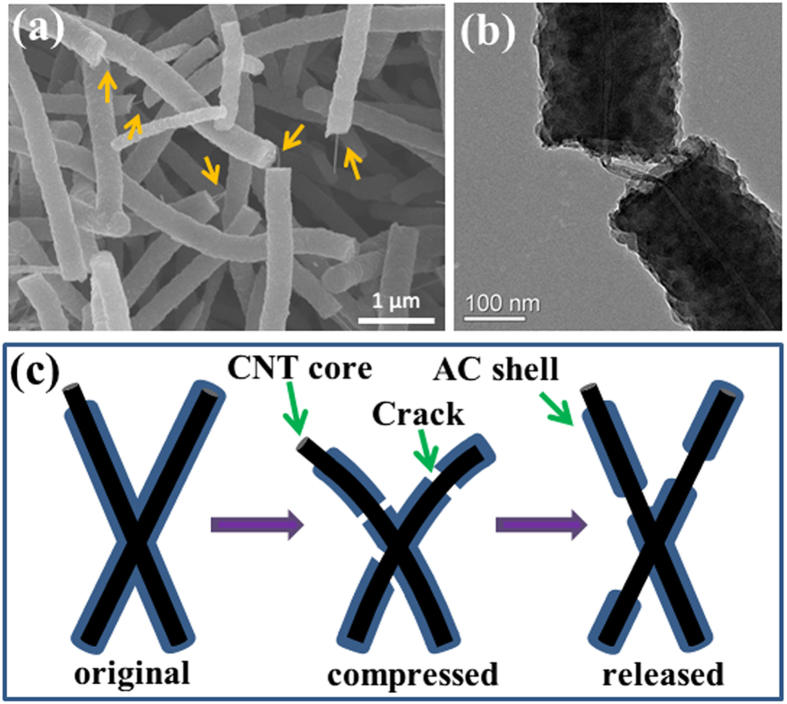
Changes in the microstructure of the samples after fatigue testing. (**a**) SEM image of the CNT solid after cyclic compression, showing the fracture of AC shells. Some CNT cores protrude out from the core-shell structures, as indicated by the arrows. (**b**) TEM image of a core-shell structure with exposed CNT at the cracked area. (**c**) Schematic showing the deformation of CNT scaffold and fracture of AC shells under cyclic compression.

So what happens in the CNT solids during cyclic compression and what is the mechanism for stress degradation? From microstructure characterization, the solids consist of a fully welded 3D CNTs due to AC deposition, resulting in significantly enhanced mechanical properties. The internal CNT network provides the solid excellent flexibility while the AC coating/welding reinforces the network rendering high compressive strength. The combination of flexible CNT cores and rigid AC shells produces a synergistic effect for achieving strong, compressible and stable porous structures. Due to its brittle nature, the relatively rigid AC shell may be fractured during deformation, as seen in SEM images after compression tests (Fig. 5a). However, the flexible CNT cores expose from the cracked area and still connect the fractured AC segments (Fig. 5b). This is completely different with CNT array coating with deposited carbon by a post-growth CVD treatment. In CNT array, the deposited carbon was in the form of additional graphitic layers, resulting in the arrays with high compressive strength and compressive resilience^23^. Although the AC-shell fracture causes stress degradation in the initial compression cycles, the embedded CNT network remains intact which is essential for maintaining the structural stability of CNT solids, as illustrated in Fig. 5c.

In summary, we demonstrated a nanoscale welding approach to fabricate reinforced 3D CNT scaffold with significant enhanced mechanical properties such as high compressive strength, flexural strength and fatigue resistance for 10^5^ cycles at high frequency (up to 10 Hz). Amorphous carbon was deposited *in situ* during CVD process, resulting in the formation of AC-CNT core-shell structures and welding at inter-CNT junctions yet still maintaining the network structure and high porosity. These CNT solids have potential applications in a wide range of fields, from applications in traditional carbon and graphite industry to complex mechanical structures and compressible functional devices, and also can work under high-frequency dynamic conditions. This *in situ* welding methodology might be useful for designing and manufacturing a series of porous materials assembled from other nanostructures (e.g. single-walled and multi-walled nanotubes, graphene, nanowires).

## Methods

### Synthesis of CNT solids

CNT solids were synthesized by CVD using ferrocene as the catalyst, and dichlorobenzene and methane as simultaneously applied carbon precursors. Ferrocene powders were dissolved in dichlorobenzene to make a precursor solution with a concentration of 0.02 g/mL, and this was injected into the CVD furnace using a syringe pump with a flow rate in the range of 0.1–0.9 mL/min. The gaseous carbon source, methane, was flowing at a rate in the range of 30–100 sccm. When the samples were synthesized at different dichlorobenzene supply rates, the methane flowing rate was set at 30 sccm. Similarly, the dichlorobenzene supply rate was set as 0.5 mL/min for different methane flowing rates. During CVD process, we adopted a reaction temperature of 850 °C, and carrier gases of argon and hydrogen at flowing rates of 2000 and 300 sccm, respectively. The height of the CNT solids can be tuned by the growth period.

### Structural characterization

The microstructure and morphology of CNTs were characterized by SEM (S-4800) and TEM (JEOL 2010HR, 200KV). Mechanical properties were tested in a single-column system (Instron 5943) at room temperature. The samples were cut into small blocks by a scalpel with sizes of about 5 × 5 × 4 (thickness) mm^3^. The top compression stage was moved down to contact the sample surface and the compression rate was set as about 1 mm/min. The fatigue properties were tested using an Instron E1000 system equipped with a 2 kN load cell and two flat-surface steel compression stages. The samples sizes are about 5 × 5 × 4 (thickness) mm^3^. All fatigue cyclic compressive tests were performed at a test frequency of 1 Hz. The compressive stress response as a function of compressive frequency was measured in the frequency range of 0.01–10 Hz.

## Additional Information

**How to cite this article**: Lin, Z. *et al.* *In-Situ* Welding Carbon Nanotubes into a Porous Solid with Super-High Compressive Strength and Fatigue Resistance. *Sci. Rep.* **5**, 11336; doi: 10.1038/srep11336 (2015).

## Supplementary Material

Supplementary Information
