# Supplementary material for: In-Situ Welding Carbon Nanotubes into a Porous Solid with Super-High Compressive Strength and Fatigue Resistance
Source: Sci Rep. 2015 Jun 11;5:11336. doi: 10.1038/srep11336 (PMC4464184; doi:10.1038/srep11336)
Supplement: Supplementary Information [file srep11336-s1.doc]

**In-Situ Welding Carbon Nanotubes into a Porous Solid with Super-High Compressive Strength and Fatigue Resistance**

Zhiqiang Lin^1^, Xuchun Gui^1^*, Qiming Gan^1^, Wenjun Chen^1^, Xiaoping Cheng^1^, Ming Liu^1^, Yuan Zhu^1^, Yanbing Yang,^2^ Anyuan Cao^2^, and Zikang Tang^1,3^

*^1^ State Key Lab of Optoelectronic Materials and Technologies, School of Physics and Engineering, Sun Yat-sen University, Guangzhou, 510275, P. R. China*

*^2^ Department of Materials Science and Engineering, College of Engineering, Peking University, Beijing 100871, P. R. China*

*^3^ Department of Physics, Hong Kong University of Science and Technology, Clear Water Bay, Kowloon, Hong Kong, China*

Corresponding authors: [guixch@mail.sysu.edu.cn](mailto:guixch@mail.sysu.edu.cn)

**Supporting Information:**

Figure S1. SEM images of the CNT solid synthesized by different dichlorobenzene supply rate. (a) 0.3 mL/min; (b) 0.5 mL/min; (c) 0.7 mL/min; (d) 0.9 mL/min.

Figure S2. TEM images of the welded CNT junctions.

Figure S3. Raman spectra of the CNT solid synthesized by different dichlorobenzene supply rate

Figure S4. SEM images of the CNT solid synthesized by different methane flowing rate. (a) 0 sccm (b) 100 sccm. (c) CNT diameter fine tuned by different methane flowing rate.

Figure S5. Measured bulk density of CNT solids depending on the carbon source supply rate.

Figure S6. Photos of the CNT solids before and after compression.

Figure S7. Mechanical curve of three-points bending. Samples size is 20.00 mm length, 7.02 mm width, and 2.22 mm height.

Table S1. the compare of the properties of CNT sponges and arrays.

| Structures | Samples | Density | Modulus | Refs. |
| --- | --- | --- | --- | --- |
| isotropic | CNT sponges | 36 mg/cm^3^ | 1 MPa | [24] |
|  | CNT sponges | 10-29 mg/cm^3^ | 1.29 MPa | [25] |
|  | Nitrogen-Doped CNT sponges | 35 mg/cm^3^ | 20 KPa | [26] |
|  | CNT sponge before coated | 10 mg/cm^3^ | 0.1 MPa | [3] |
|  | AC coated CNT sponge | 363 mg/cm^3^ | 107 MPa | Present work |
|  |  |  |  |  |
| anisotropy | CNT arrays | 200 mg/cm^3^ | 138.5 MPa | [38] |
|  | CNT arrays | 80 mg/cm^3^ | 120 MPa | [57] |
|  | CNT arrays | 114 mg/cm^3^ | 13 MPa | [23] |
|  | CNT array (under compression parallel to the nanotube axis) | 120 mg/cm^3^ | 0.55 MPa | [20] |
|  | CNT array (under compression normal to the nanotube axis) |  | 1.09 MPa | [20] |
|  | CNT arrays | / | 1.0 MPa | [13] |
|  | CNT arrays | / | 50 MPa | [15] |
|  | CNT arrays |  | 50 MPa | [37] |
|  | CNT arrays before coated | / | 200 MPa | [21] |
|  | a-SiC coated CNT arrays |  | 125 GPa |  |
|  | CNT arrays before coated | / | 14 MPa | [22] |
|  | Al_2_O_3_ coated CNT arrays | 1000 mg/cm^3^ | 20 GPa |  |
|  | Carbon coated CNT arrays | 950 mg/cm^3^ | 20GPa | [16] |
